# Synthesis and Properties of a Novel Levulinic Acid-Based Environmental Auxiliary Plasticizer for Poly(vinyl chloride)

**DOI:** 10.3390/polym16030361

**Published:** 2024-01-29

**Authors:** Zeyu You, Min Yu, Renli Fu, Xiaoan Nie, Jie Chen

**Affiliations:** 1 Key Laboratory of Biomass Energy and Material, Institute of Chemical Industry of Forest Products, Chinese Academy of Forestry, Nanjing 210042, China; 2 College of Materials Science and Technology, Nanjing University of Aeronautics and Astronautics, Nanjing 210016, China

**Keywords:** renewable origin, ketal, methyl eleostearate, plasticizer, poly(vinyl chloride)

## Abstract

Herein, a bio-based plasticizer ketalized tung oil butyl levulinate (KTBL) was developed using methyl eleostearate, a derivative of tung oil, and butyl levulinate. KTBL can be used as an auxiliary plasticizer to partially replace traditional plasticizer. The plasticizer has a ketone structure, an ester base, and a long linear chain. It was mixed with dioctyl phthalate (DOP), and the effect of the plasticizer KTBL as an auxiliary plasticizer on the plasticization of poly(vinyl chloride) (PVC) was studied. Their compatibility and plasticizing effect were evaluated using dynamic–mechanical thermal analysis (DMA), mechanical property analysis, and thermogravimetric analysis (TGA). The results demonstrate that when the KTBL to DOP ratio is 1:1, the blended sample with KTBL exhibits superior mechanical performance compared to pure DOP, resulting in an increased elongation at break from 377.47% to 410.92%. Moreover, with the increase in KTBL content, the durability is also significantly improved. These findings suggest that KTBL can serve as an effective auxiliary plasticizer for PVC, thereby reducing the reliance on DOP.

## 1. Introduction

Polyvinyl chloride (PVC), the third-largest plastic in terms of global production, exhibits an annual output exceeding 40 million tons and finds extensive application across diverse sectors including food packaging, automotive manufacturing and bio-medicine [1,2,3]. Its popularity can be attributed to its excellent mechanical properties, chemical corrosion resistance, and flame retardancy due to the presence of repeated C-Cl polar bonds. However, pure PVC faces challenges during processing due to its high melting viscosity and lack of flexibility [4,5,6]. To overcome these limitations, additives like stabilizers and plasticizers are incorporated into PVC formulations to tailor their properties accordingly. Plasticizers belong to a category of additives that enhance the processing properties and product flexibility of polymer materials. In the case of PVC, an ideal plasticizer should possess both polar groups and C-Cl polar bonds while also including non-polar or weakly polar groups that disrupt intermolecular interactions between chains [7]. By incorporating suitable plasticizers into PVC formulations, the processability of PVC can be significantly improved. These plasticizers act as molecular lubricants within the polymer matrix by reducing intermolecular forces between polymer chains. This results in lower melt viscosity and enhanced flow characteristics during processing. Furthermore, plasticizers also play a crucial role in improving the flexibility of PVC products [8]. The incorporation of non-polar or weakly polar groups disrupts the regular packing arrangement of polymer chains in solid-state form. As a result, it increases chain mobility and allows for greater flexibility without compromising other desirable properties [9]. The choice of an appropriate plasticizer depends on specific application requirements such as desired mechanical strength, thermal stability, and compatibility with other additives or fillers present in the formulation. Different types of plasticizers offer varying degrees of performance enhancement for PVC-based materials.

Currently, a wide range of chemicals are utilized as plasticizers, including phthalates, fatty esters, benzoate esters, phosphate esters, polyesters, and various other co-plasticizer materials. Among these options, phthalate plasticizers derived from C_8_-C_10_ alcohols (particularly DOP) are extensively employed due to their versatility and cost-effectiveness [10]. However, unfortunately, phthalate plasticizers have a tendency to migrate and leach during usage. This not only diminishes the performance of plastics [11,12,13], but also poses significant risks to human health and the environment [14,15,16,17]. Consequently, regulations and restrictions on the use of phthalates have been implemented in numerous countries and regions such as the United States [18], Canada [19], and Europe [20], particularly for plastic products that come into close contact with humans like children’s toys, food packaging, and medical devices. Therefore, there is an urgent need for the development of a new generation of plasticizers that can replace DOP [21,22]. Biomass is a sustainable and non-toxic source, offering a wide range of biomass-derived chemical raw materials with diverse convertible functional groups, including double bonds, hydroxyl groups, ester groups, carboxyl groups, ether groups, carbonyl groups, and epoxy groups. This abundance provides abundant opportunities for molecular modification in the design and synthesis of innovative bio-based plasticizers. Researchers have successfully synthesized a diverse range of structurally novel environmentally friendly plasticizers through reactions such as esterification, etherification, and epoxidation [23,24,25]. Many of these exhibit promising potential to replace conventional phthalate-based plasticizers, while certain bio-based alternatives have even progressed to the stage of commercialization, including epoxy soybean oil [26,27], citrate esters [28,29], and castor oil [30]. However, the identification and synthesis of novel compounds continue to pose a formidable challenge. To date, no additive has been discovered that can rival traditional phthalates in terms of both high-performance capabilities and cost-effectiveness. Consequently, the development of an exceptionally efficient bio-based plasticizer is considered a promising area for future investigation [31].

China dominates the global production of tung oil, accounting for 80% of the total output. Approximately 80% of tung oil derivatives consist of glycerol esters of octadecyl-conjugated triene fatty acids, known as tung acid triol esters. The molecular structure of tung oil contains various active reaction sites such as conjugated double bonds, ester bonds, allyl groups, and three long flexible fatty acid chains. As a result, a wide range of functional tung oil derivatives have been derived and extensively utilized in fields including curing agents, ink production, paint manufacturing, and lubricant formulation, among others [32]. However, the utilization of tung oil as a raw material for synthesizing plasticizers is relatively uncommon, thus indicating significant development potential for utilizing tung-oil-based plasticizers. Moreover, levulinic acid has gained considerable attention due to its biomass origin and comparatively low cost [33,34,35]. It serves as an intermediate compound in synthetic product manufacturing and finds applications across diverse sectors including polymer additives production, surfactant fuel formulation, the fragrance industry, solvent usage, and the pharmaceuticals sector. The presence of both carboxyl and ketone groups in levulinic acid enables the preparation of these compounds. Recent research reports have demonstrated that certain plasticizers containing ketal groups exhibit superior plasticizing performance in PVC compared to DOP due to the interaction between the ketal group and PVC [36]. Based on this discovery, a novel bio-based plasticizer (KTBL) was synthesized by employing camphoric methyl eleostearate and acetyl propionic acid butyl ester as raw materials through amide formation and ketalization. This innovative plasticizer possesses both ketal and ester groups, along with an alkyl long chain, making it suitable for utilization as an auxiliary additive to reduce reliance on phthalate-based plasticizers.

## 2. Materials and Methods 

### 2.1. Materials

Methyl eleostearate (ME, lab-made), diethanolamine (DEA, AR, Macklin, Shanghai, China), potassium hydroxide (KOH, Macklin), butyl levulinate (98%, Aladdin, Shanghai, China), and ptoluene sulfonic acid monohydrate (PTSA·H_2_O, 98.5%, Aladdin) were used for the synthesis of plasticizers. Potassium carbonate (GR for analysis, Macklin) and ethyl acetate (analytical reagent grade, Aladdin) were utilized during the extraction to purify the plasticizers. Polyvinyl chloride (PVC, K value 65–67, Tianjin Dagu Chemical Co., Ltd., Tianjin, China), dichloroethane (98%, Aladdin), and tetrahydrofuran (THF, AR; Sinopharm Chemical Reagent, Shanghai, China) were utilized as the solvent and film-forming polymer during solution casting of films with or without plasticizers. Dioctyl phthalate (DOP, ≥98%, Aladdin) was selected as the reference plasticizer. Ethanol (AR, Sinopharm Chemical Reagent), acetic acid glacial (AR, Macklin), and water were used to prepare simulants for the migration measurements. All chemicals were used as received.

### 2.2. Synthesis of Plasticizer 

**Figure 1:**
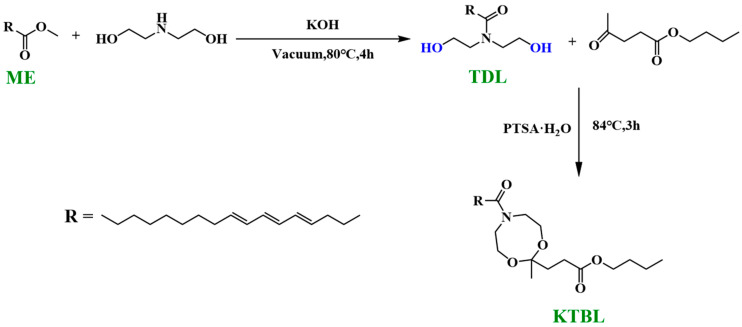
Synthesis of plasticizer KTBL.

The specific synthesis route of the plasticizer is shown in Figure 1. Tung oil–diethanolamine (TDL) was prepared via amidation of ME with DEA. Then, TDL was further modified via ketalization with butyl 4-oxopentanoate to obtain the plasticizer KTBL.

#### 2.2.1. Synthesis of Tung-Oil-Based Diethanolamine

The amidation reaction between ME and DEA was conducted following the established protocols [37]. A round-bottom flask containing ME (292 g, 1.0 mol), DEA (117.17 g, 1.11 mol), and KOH (2.05 g, 0.5 wt %) was subjected to vacuum stirring at 80 °C for 4 h to facilitate the amidation reaction (no gas evolution occurred during the reaction). Subsequently, the resulting product was extracted with ethyl acetate and washed with saturated NaCl solution to remove excess DEA. Finally, the organic phase was dried over MgSO_4_, filtered, and subjected to vacuum distillation to obtain the TDL product.

#### 2.2.2. Synthesis of Ketalized Tung Oil Butyl Levulinate

In a round-bottom flask, TDL (35 g, 0.1 mol) was dissolved in dichloroethane (300 mL), followed by the addition of ethyl acetoacetate (20 g, 0.1 mol). PTSA (1.4 g, 0.008 mol) was added as a catalyst. The mixture was stirred and refluxed at 84 °C for 4 h under a nitrogen atmosphere before being cooled to room temperature. Subsequently, the product was neutralized with saturated potassium carbonate solution and the organic phase was separated and filtered. Finally, the filtrate underwent vacuum distillation to obtain a pale red oil. 

### 2.3. Preparation of PVC Films

**Figure 2:**
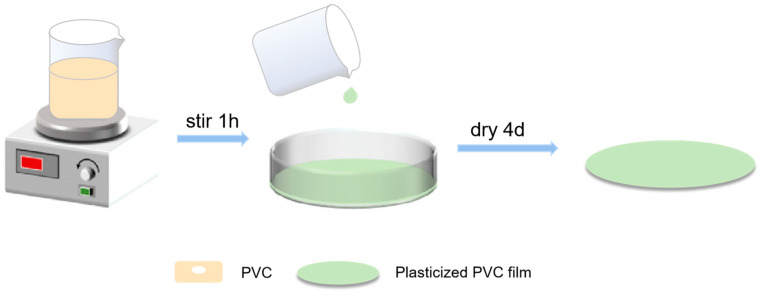
Preparation of plasticized PVC samples.

Polyvinyl chloride films containing various plasticizers were prepared using a solution method (Figure 2), with the specific plasticizer formulations provided in Table 1. A mixture of polyvinyl chloride (10 g) and plasticizer (4 g) was dissolved in 150 mL of THF, then vigorously stirred at 55 °C for 1 h to achieve a homogeneous solution. The resulting transparent mixture was then cast onto a Petri dish (diameter: 19 cm) and air-dried at room temperature for 48 h, followed by further drying at 60 °C in an oven for another 48 h to ensure complete removal of residual THF. The resulting polyvinyl chloride film exhibited a thickness ranging from 0.5 to 0.6 mm. 

### 2.4. Characterizations and Measurements

The Fourier infrared spectra of the compounds were obtained by measuring them with an infrared spectrometer (Nicolet iS50-Thermo Fisher Scientific, Waltham, MA, USA), which had a scanning range spanning from 4000 to 400 cm^−1^ and a resolution of 4 cm^−1^, all conducted under total reflection. The tensile properties were evaluated using the LD24.304 microcomputer-controlled electronic universal testing machine (Lishi (Shanghai) Instruments Co., Ltd, Shanghai, China) according to GB/T 1040.1-2006 (China) [23]. Testing was performed at a temperature of 25 °C, employing a stretching rate of 20 mm/min, and results from five parallel samples were averaged.

The glass transition temperature (T_g_) of the sample was measured in tensile mode using a Q800 dynamic thermomechanical analyzer (TA Instruments, New Castle, DE, USA). The test range spanned from −60 to 80 °C, with a heating rate of 3 °C·min^−1^ and a test frequency of 1 Hz. The thermogravimetric behavior of the samples was analyzed using a TG209F1 thermogravimetric analyzer (Netzsch Instrument Corp., Bavaria, Germany) under a N_2_ atmosphere, employing a heating rate of 10 °C·min^−1^ within a temperature range from 25 to 600 °C.

According to ASTM 1239-98 [23], PVC samples (25 × 25 × 0.5 mm) were immersed in the distilled water and ethanol solution at a concentration of 10%, the acetic acid solution at a concentration of 30%, and petroleum ether at room temperature controlled at 25 °C for a duration of 24 h. After removal from each solution, they were rinsed with running water, thoroughly dried, and subsequently placed in an oven set at 30 °C for an additional period lasting another day (24 h). Changes in quality before and after impregnation were recorded accordingly. The calculation formula is as follows:

(1) $$\mathrm{Weight}\;\mathrm{loss}\left(\%\right)=\frac{W_{1}-W_{2}}{W_{1}}\times 100\%$$

where *W*_1_ and *W*_2_ represent the initial and final weight of the sample, respectively.

Volatility testing was conducted in accordance with ISO 176:2005 [23]. The PVC sample (25 × 25 × 0.5 mm) is positioned at the base of a metallic container and covered with activated carbon. Subsequently, the metal container is placed within an oven or thermostatic water bath set at a temperature of 70 ± 1 °C for a duration of 24 h, followed by gradual cooling to room temperature using a dryer. Any residue present on the surface of the activated carbon should be meticulously removed, while documenting any observed alterations in quality pre and post test.

The PVC sample was placed between two pieces of filter paper and subjected to a temperature of 60 °C in an oven for a duration of 48 h. The difference in mass observed before and after the use of the filter paper indicates the quantity of sample exudation.

## 3. Results and Discussion

### 3.1. Synthesis and Characterization of Plasticizers

**Figure 3:**
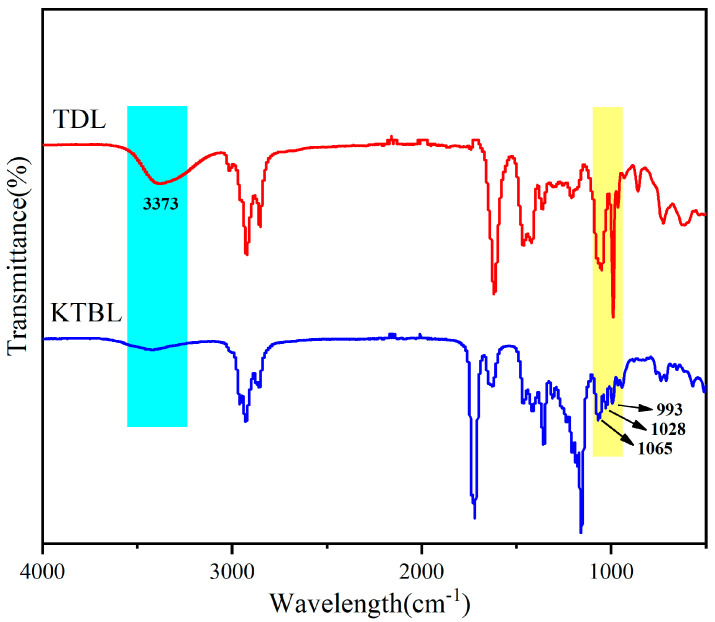
FTIR spectra of TDL and KTBL.

To further evaluate the conformity of the final product’s structure, Fourier-transform infrared spectroscopy (FTIR) analysis was performed. The obtained results are presented in Figure 3. Within the FTIR spectrum of TDL, a distinct absorption band at 3373 cm^−1^ was observed, which corresponds to the stretching vibration peak of terminal hydroxyl groups on TDL. However, upon analyzing the KTBL spectrum, a significant reduction in hydroxyl intensity was noticed. This decrease suggests that these hydroxyl groups have undergone a reaction and their content has substantially decreased. Additionally, prominent absorption peaks were observed at 2922 and 2852 cm^−1^ in the KTBL spectrum, indicating the presence of methyl and methylene groups, respectively. Furthermore, a strong absorption band at 1719 cm^−1^ confirms the existence of ester bonds within this spectrum. Moreover, characteristic triad peaks representing ketal groups were identified at 1065, 1028, and 993 cm^−1^ (–C–O–C–C–O–C–) within the KTBL spectrum [38]. These findings strongly support our initial expectations regarding product structure as they demonstrate that ketone groups have indeed reacted with diols to form ketal groups. In conclusion, through FTIR analysis we have successfully confirmed that our anticipated product structure has been achieved by observing changes in hydroxyl intensity and identifying specific functional group vibrations within both TDL and KTBL spectra.

### 3.2. Dynamic Thermomechanical Analysis of PVC Samples

**Figure 4:**
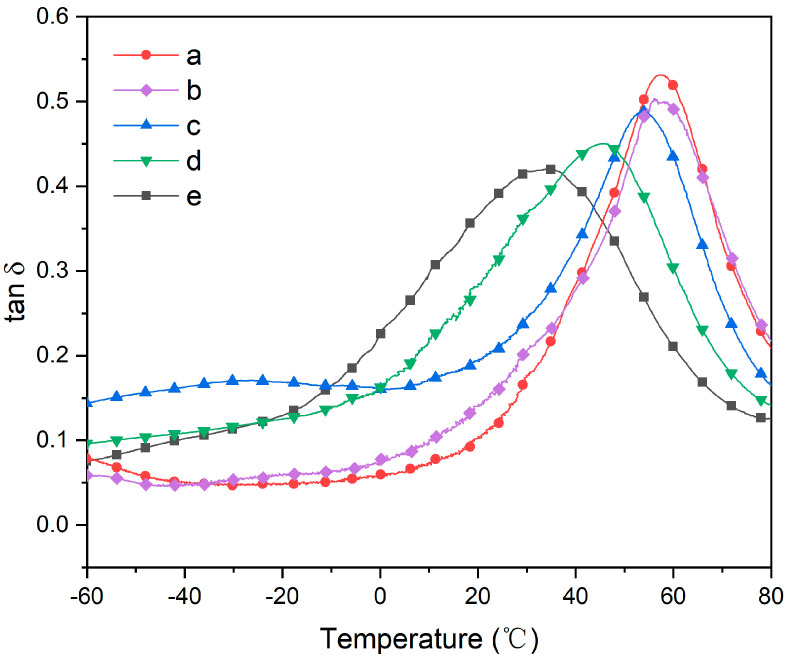
DMA curve of PVC sample.

**Figure 5:**
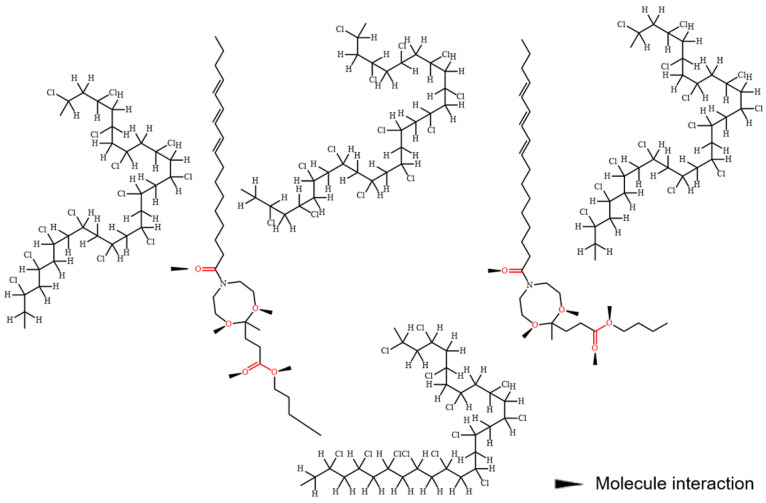
The potential interactions between KTBL and PVC molecules.

The dynamic mechanical analysis curves of different PVC samples are presented in Figure 4, with detailed data provided in Table 2. It is well known that the peak temperature on the tan δ curve corresponds to the glass transition temperature (T_g_). The T_g_ values for PVC-a, PVC-b, PVC-c, PVC-d, and PVC-e are determined as 56.55 °C, 54.95 °C, 53.76 °C, 46.00 °C, and 34.91 °C, respectively, whereas pure PVC typically exhibits a T_g_ value within the range of 80–90 °C. Notably, KTBL as a plasticizer demonstrates a relatively limited ability to reduce the polymer’s T_g_ compared to DOP. Despite variations in plasticizer content between samples such as PVC-a and PVC-b, their respective T_g_ values do not exhibit significant differences until sample PVC-d, where a noticeable decrease in T_g_ becomes apparent. This suggests that its plasticizing effect remains modest at low DOP concentrations and only becomes prominent upon reaching a certain concentration level. According to the plasticizer theory, the incorporation of plasticizer molecules into the PVC matrix facilitates their long-chain structure to intercalate between polymer chains, thereby increasing the spacing between chains and enhancing the free volume among polymer molecules, ultimately promoting flexibility in the polymer. Additionally, polar groups present in plasticizers can counteract intermolecular interactions among polymer chains through van der Waals forces with polar groups in polymers, leading to improved PVC chain mobility and a reduced glass transition temperature [9,39]. The schematic diagram illustrating its mechanism is presented in Figure 5. KTBL not only exhibits interactions between the ketal groups and ester groups but also possesses linear long chains that can be intercalated between polymer chains to enhance the polymer’s free volume. However, it did not demonstrate the expected performance in compatibility tests due to KTBL’s higher molecular weight (505 g/mol) compared to DOP (391 g/mol), resulting in a lower number of polar groups per unit mass and consequently reducing PVC’s plasticity.

### 3.3. Thermogravimetric Analysis of PVC Samples

**Figure 6:**
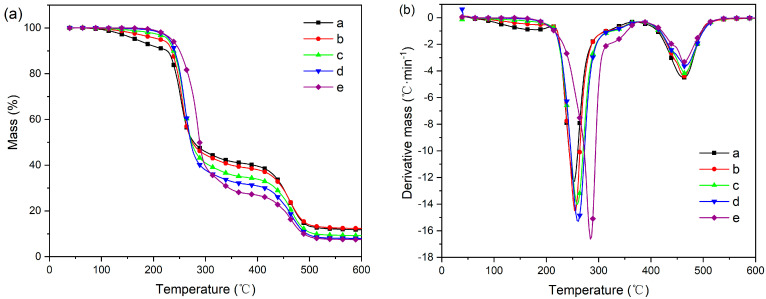
TG (**a**) and DTG (**b**) curve of PVC samples.

Thermogravimetric analysis (TGA) is a widely employed technique for investigating the thermal stability and degradation behavior of materials. In this study, TGA was conducted on PVC samples to explore their thermal properties. The acquired data, as depicted in Figure 6 and Table 2, offer valuable insights into the degradation process of PVC. Two crucial parameters were examined: mass loss at 50% (T_50_) and maximum weight loss temperature rate (T_P1_ and T_P2_). These parameters aid in comprehending the extent of degradation and the temperature range within which it occurs. Compared with other plastics, PVC demonstrates a lower thermal degradation temperature due to its inherent composition comprising thermally unstable structural segments and defects. The presence of these elements renders PVC more susceptible to heat-induced deterioration. A key contributing factor to the thermal instability of PVC is its chlorine content. During degradation, the so-called “unstable” chlorine atoms undergo dehydrochlorination reactions, resulting in the formation of conjugated polyene sequences within the polymer chain. This process generates a zipper-like structure that further facilitates degradation [40,41]. Consequently, PVC experiences a decline in its physical and mechanical properties as a consequence of this chemical transformation; it becomes weaker, less flexible, and more prone to brittleness or embrittlement over time. Understanding the thermal behavior of PVC holds significant importance for various applications where heat resistance plays an essential role.

The thermal degradation process of PVC, as depicted in Figure 6a, can be divided into two distinct stages. The first stage occurs within the temperature range of 200–400 °C and is primarily attributed to the dehydrochlorination of PVC molecular chains, resulting in the formation of conjugated polyolefin structures. This process is facilitated by the generation of HCl, which acts as a catalyst. It is noteworthy that this initial stage exhibits the highest rate of thermal decomposition, with approximately 60–70% of the total weight loss occurring within this temperature range. Moving on to the second stage, which takes place above 400 °C, it mainly involves C-C fracture within the conjugated polyolefin structure and cyclization reactions leading to aromatic compound formation. According to the results depicted in Figure 6a, when compared with the dual-component plasticizer, the single-component plasticizer demonstrates a higher T_50_ value. The difference can be attributed to the relatively inferior thermal stability of KTBL compared to DOP; however, at lower concentrations, the plasticizing effect of DOP is not significant. Consequently, pure KTBL and DOP as individual plasticizers exhibit favorable T50 values. Nevertheless, due to the limited concentration of DOP in binary plasticizers, its plasticizing effect cannot be fully harnessed, resulting in a lower T50 value for the binary plasticizer. By analyzing the DTG curves illustrated in Figure 6b, we observe that T_p1_ and T_p2_ occur at temperatures ranging from 252 to 282 °C and 463 to 465 °C, respectively. These peak temperatures represent crucial points where significant changes in thermogravimetric rates are observed during both stages of thermal degradation. The T_P1_ value gradually increases with the decrease in KTBL content, attributed to the inherently low thermal decomposition temperature of KTBL. Interestingly, when examining how KTBL content influences these processes, it becomes apparent that its impact is more pronounced during the second stage rather than in the initial phase. This suggests that KTBL plays a relatively minor role in promoting dehydrochlorination but exerts greater influence on subsequent reactions involving C-C fracture and aromatic compound generation. Furthermore, an analysis of Table 2 reveals an intriguing trend regarding char yield—a measure indicating residual solid material after thermal degradation. As shown, there is a gradual increase in char yield from film PVC-d (7.62%) to film PVC-a (11.22%) with increasing KTBL content. The aforementioned observation suggests that KTBL demonstrates reduced thermal degradation at elevated temperatures, leading to an augmented char residue ratio.

### 3.4. Tensile Properties of PVC Samples

Mechanical properties play a pivotal role when assessing the plasticizing effect of PVC plasticizers. Table 3 presents specific data on the elongation at break and tensile strength of each PVC sample. Pure PVC typically exhibits an elongation at break of approximately 40% and a tensile strength ranging from 50 to 80 MPa. Based on the tabulated data, it can be observed that all plasticizers effectively enhance the flexibility of PVC while reducing its rigidity. The elongation at break of the PVC samples initially increases and then decreases with decreasing KTBL content, reaching a peak value of 410.92% when the ratio of KTBL to DOP is 1:1. Similarly, the tensile strength follows a similar trend, reaching a peak value of 25.35 Mpa when the ratio of KTBL to DOP is 1:3. Samples PVC-c and PVC-d demonstrate superior overall mechanical properties compared to the other samples due to synergistic effects between DOP and KTBL. Firstly, through van der Waals forces, polar groups (such as ester groups, ketone groups, amide groups, and benzene rings) in plasticizers lubricate the non-crystalline region of PVC molecules by interacting with repetitive polar bonds in PVC molecules, thus reducing the polarity between them. The long alkyl chain structure present in KTBL can penetrate into crystalline regions within PVC chains to improve product flexibility [9]. In addition to the inherent interaction between plasticizers and PVC molecules, as the concentration of plasticizers gradually increases, the interplay among different plasticizers becomes increasingly prominent. The interaction between KTBL and DOP enhances the plasticizing efficiency of both substances, with the strongest effect observed when their ratio is close [39]. This phenomenon also elucidates why a 1:1 mixture of these two plasticizers exhibits optimal performance. In conclusion, KTBL can serve as an auxiliary plasticizer in combination with DOP to enhance plasticization efficiency.

### 3.5. Durability Analysis of PVC Samples

**Figure 7:**
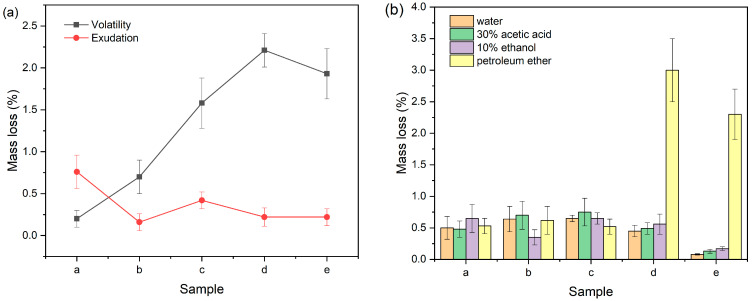
The weight loss in (**a**) exudation, volatility, and (**b**) extraction testing of various PVC films.

After exposure to high temperatures for a specific duration, plastic products undergo a migration of low-molecular-weight plasticizers onto the surface and surrounding environment, thereby impacting the material’s performance. This phenomenon is of great importance in industries where plastics are extensively used, such as packaging and construction. In Figure 7a, we observe the volatility and migration losses of various PVC samples. Notably, an increase in KTBL content resulted in decreased volatility loss from 1.97% to 0.20%. This reduction can be attributed to KTBL’s higher molecular weight compared to DOP, resulting in lower volatility loss due to better retention within the polymer matrix, even under high-temperature conditions. Additionally, regardless of variations in KTBL content, all samples exhibited consistently low migration losses with no significant differences observed. These findings have practical implications for manufacturers and researchers seeking to improve plastic product performance under elevated temperature conditions by selecting appropriate plasticizers with higher molecular weights like KTBL, instead of traditional ones like DOP.

Based on Figure 7b, extraction losses of PVC samples with different formulations in distilled water, 10% ethanol, 30% acetic acid, and petroleum ether were observed. Among them, PVC-a, PVC-b, and PVC-c exhibited relatively low levels of extraction losses in each organic solvent without significant differences between them. In contrast, PVC-d and PVC-e were easily extracted by petroleum ether due to the propensity of DOP to be extracted in this solvent, which reduces the plastic’s performance. This is because KTBL has a higher relative molecular weight compared to DOP and demonstrates greater resistance to extraction in other solutions. Therefore, even when exposed to certain organic solvents, the combination of these two plasticizers can maintain higher durability.

## 4. Conclusions

In summary, a bio-based plasticizer named KTBL, containing ketal groups, straight-chain ester groups, and long alkyl chains, was synthesized and blended with DOP to investigate its efficacy as an auxiliary plasticizer. The structure of the synthesized plasticizer was confirmed through Fourier-transform infrared spectroscopy (FTIR). All plasticizers significantly reduced the glass transition temperature (T_g_) of PVC. The combination of KTBL and DOP can significantly enhance the thermal stability of PVC, surpassing the impact of the pure KTBL plasticizer. Furthermore, PVC films incorporating both types of plasticizers exhibited improved mechanical properties compared to those using only one type. Moreover, an increase in KTBL content led to decreased volatility loss and extraction rate, indicating a significant improvement in the stability of the plasticizer within plastic products. Maintaining a 1:1 content ratio between KTBL and DOP resulted in an optimal comprehensive performance since high concentrations of plasticizers promote interactions that enhance their effectiveness on PVC. Therefore, this novel design of a plasticizer made from methyl stearate and butyl acrylate can serve as an auxiliary additive to partially replace DOP while reducing the environmental impact and presenting promising application prospects.

## Figures and Tables

**Table 1:** Composition of PVC blends.

Sample	PVC (g)	KTBL (g)	DOP (g)
PVC-a	10	4	0
PVC-b	10	3	1
PVC-c	10	2	2
PVC-d	10	1	3
PVC-e	10	0	4

**Table 2:** Thermogravimetric data of PVC samples.

Sample	T_g_ (°C)	T_50_ (°C)	T_p1_ (°C)	T_p2_ (°C)	Char Yield (%)
PVC-a	56.55	277.8	252.3	463.2	11.22
PVC-b	54.95	274.5	255.2	462.8	11.87
PVC-c	53.76	272.1	259.3	464.6	8.90
PVC-d	46.00	271.5	260.6	465.2	7.62
PVC-e	34.91	288.9	285.9	464.1	7.25

**Table 3:** Tensile properties of PVC samples.

Sample	Percent Elongation (%)	Tensile Strength (MPa)
PVC-a	384.93 ± 17.25	19.32 ± 0.14
PVC-b	379.25 ± 17.00	21.78 ± 1.18
PVC-c	410.92 ± 16.36	23.16 ± 1.46
PVC-d	403.72 ± 10.64	25.35 ± 2.36
PVC-e	377.47 ± 12.43	23.03 ± 1.03

## Data Availability

The original contributions presented in the study are included in the article, further inquiries can be directed to the corresponding author.

## References

[1] Ali M., Lu Y., Ahmed S., Khanal S., Xu S. (2020). Effect of Modified Cardanol as Secondary Plasticizer on Thermal and Mechanical Properties of Soft Polyvinyl Chloride. ACS Omega.

[2] Allan Stahl G. (1982). Polymer Chemistry: An Introduction. J. Chem. Educ..

[3] Navarro R., Perrino M., García C., Elvira C., Gallardo A., Reinecke H. (2016). Opening New Gates for the Modification of PVC or Other PVC Derivatives: Synthetic Strategies for the Covalent Binding of Molecules to PVC. Polymers.

[4] Ciacci L., Passarini F., Vassura I. (2016). The European PVC Cycle: In-Use Stock and Flows. Resour. Conserv. Recycl..

[5] Esckilsen B. (2008). Global PVC Markets: Threats and Opportunities. Plast. Addit. Compd..

[6] Cappucci L.R. (2009). PVC: A Vital and Sustainable Resource. Plast. Addit. Compd..

[7] Titow W.V. (1990). PVC Plastics: Properties, Processing, and Applications.

[8] Liu H. (2011). Mechanism of PVC Plasticization and PVC-Plasticizer Interaction. Shanxi Chem. Ind..

[9] Groover M.P. (2010). Fundamentals of Modern Manufacturing: Materials, Processes, and Systems.

[10] Demir A.P.T., Ulutan S. (2013). Migration of Phthalate and Non-Phthalate Plasticizers out of Plasticized PVC Films into Air. J. Appl. Polym. Sci..

[11] Bonini M., Errani E., Zerbinati G., Ferri E., Girotti S. (2008). Extraction and Gas Chromatographic Evaluation of Plasticizers Content in Food Packaging Films. Microchem. J..

[12] Kastner J., Cooper D.G., Mari M., Dodd P., Yargeau V. (2012). Aqueous Leaching of Di-2-Ethylhexyl Phthalate and “Green” Plasticizers from Poly(Vinyl Chloride). Sci. Total Environ..

[13] Nagorka R., Conrad A., Scheller C., Süssenbach B., Moriske H.J. (2011). Diisononyl 1,2-Cyclohexanedicarboxylic Acid (DINCH) and Di(2-Ethylhexyl) Terephthalate (DEHT) in Indoor Dust Samples: Concentration and Analytical Problems. Int. J. Hyg. Environ. Health.

[14] Bui T.T., Giovanoulis G., Cousins A.P., Magner J., Cousins I.T., Wit C.A.D. (2016). Human Exposure, Hazard and Risk of Alternative Plasticizers to Phthalate Esters. Sci. Total Environ..

[15] Bustamante-Montes L.P., Hernández-Valero M.A., Flores-Pimentel D., García-Fábila M., Amaya-Chávez A., Barr D.B., Borja-Aburto V.H. (2013). Prenatal Exposure to Phthalates Is Associated with Decreased Anogenital Distance and Penile Size in Male Newborns. J. Dev. Orig. Health Dis..

[16] Colón I., Caro D., Caro D. (2000). Identification of Phthalate Esters in the Serum of Young Puerto Rican Girls with Premature Breast Development. Environ. Health Perspect..

[17] Piché C.D., Sauvageau D., Vanlian M., Erythropel H.C., Robaire B., Leask R.L. (2012). Effects of Di-(2-Ethylhexyl) Phthalate and Four of Its Metabolites on Steroidogenesis in MA-10 Cells. Ecotoxicol. Environ. Saf..

[18] Flaherty E. (2008). Consumer Product Safety Improvement Act of 2008. Loyola Consum. Law Rev..

[19] Canada E., Canada H. (1994). Priority Substances List Assessment Report: Bis (2-Ethylhexyl) Phthalate.

[20] Directive 2005/84/EC of the European Parliament and of the Council of 14 December 2005. https://eur-lex.europa.eu/legal-content/EN/TXT/?uri=celex%3A32005L0084.

[21] Li W., Qin J., Wang S., Han D., Xiao M., Meng Y. (2018). Macrodiols Derived from CO2-Based Polycarbonate as an Environmentally Friendly and Sustainable PVC Plasticizer: Effect of Hydrogen-Bond Formation. ACS Sustain. Chem. Eng..

[22] Erythropel H.C., Marie M., Cooper D.G. (2012). Designing Green Plasticizers: Influence of Molecular Geometry on Biodegradation and Plasticization Properties. Chemosphere.

[23] Han Y., Zhang C., Yang Y., Weng Y., Ma P., Xu P. (2023). Epoxidized Isosorbide-Based Esters with Long Alkyl Chains as Efficient and Enhanced Thermal Stability and Migration Resistance PVC Plasticizers. Polym. Test..

[24] Rodrigues F.M.S., Tavares I., Aroso R.T., Dias L.D., Domingos C.V., De Faria C.M.G., Piccirillo G., Maria T.M.R., Carrilho R.M.B., Bagnato V.S. (2023). Photoantibacterial Poly(Vinyl)Chloride Films Applying Curcumin Derivatives as Bio-Based Plasticizers and Photosensitizers. Molecules.

[25] Duan X., Chen H., Liu H., Chen M., Chen S., Gao J. (2022). A Citric Acid/Cysteine Based Bioadditive for Plasticization and Enhancing UV Shielding of Poly(Vinyl Chloride). Polym. Int..

[26] Chen J., Li X., Wang Y., Li K., Huang J., Jiang J., Nie X. (2016). Synthesis and Application of a Novel Environmental Plasticizer Based on Cardanol for Poly(Vinyl Chloride). J. Taiwan Inst. Chem. Eng..

[27] Jia P.Y., Zhang L.H., Zhou Y.H. (2016). Green Plasticizers Derived from Soybean Oil for Poly(Vinyl Chloride) as a Renewable Resource Material. Korean J. Chem. Eng..

[28] Jia P., Hu L., Feng G., Bo C., Zhang M., Zhou Y. (2017). PVC Materials without Migration Obtained by Chemical Modification of Azide-Functionalized PVC and Triethyl Citrate Plasticizer. Mater. Chem. Phys..

[29] Ljungberg N., Wesslén B. (2003). Tributyl Citrate Oligomers as Plasticizers for Poly (Lactic Acid): Thermo-Mechanical Film Properties and Aging. Polymer.

[30] Mhaske S., Satavalekar S.D., Savvashe P. (2016). Triester-Amide Based on Thiophene and Ricinoleic Acid as an Innovative Primary Plasticizer for Poly(Vinyl Chloride). RSC Adv..

[31] Sinisi A., Degli Esposti M., Toselli M., Morselli D., Fabbri P. (2019). Biobased Ketal−Diester Additives Derived from Levulinic Acid. ACS Sustain. Chem. Eng..

[32] Yang X., Li S., Xia J., Song J., Li M. (2015). Novel Renewable Resource-Based UV-Curable Copolymers Derived from Myrcene and Tung Oil: Preparation, Characterization and Properties. Ind. Crops Prod..

[33] Bernhard Y., Pagies L., Pellegrini S., Bousquet T., Favrelle A., Pelinski L., Gerbaux P., Zinck P. (2019). Synthesis of Levulinic Acid Based Poly(Amine-Co-Ester)s. R. Soc. Chem..

[34] Bozell J.J., Moens L., Elliott D., Wang Y., Neuenscwander G., Fitzpatrick S., Bilski R., Jarnefeld J. (2000). Production of Levulinic Acid and Use as a Platform Chemical for Derived Products. Resour. Conserv. Recycl..

[35] Isikgor F.H., Becer C.R. (2015). Lignocellulosic Biomass: A Sustainable Platform for the Production of Bio-Based Chemicals and Polymers. Polym. Chem..

[36] Zhu H., Yang J., Wu M., Wu Q., Liu J., Zhang J. (2021). Effect of Ketal Group in Castor Oil Acid-based Plasticizer on the Properties of Poly(Vinyl Chloride). J. Appl. Polym. Sci..

[37] Xiao L., Li W., Liu Z., Zhang K., Li S., Wang Y., Chen J., Huang J., Nie X. (2022). Tung Oil-Derived Epoxy Vitrimers with High Mechanical Strength, Toughness, and Excellent Recyclability. ACS Sustain. Chem. Eng..

[38] Socrates G. (2001). Infrared and Raman Characteristic Group Frequencies: Tables and Charts.

[39] Godwin A.D. (2011). Plasticizers. Applied Plastics Engineering Handbook.

[40] Jia P., Hu L., Shang Q., Wang R., Zhang M., Zhou Y. (2017). Self-Plasticization of PVC Materials via Chemical Modification of Mannich Base of Cardanol Butyl Ether. ACS Sustain. Chem. Eng..

[41] Yu J., Sun L., Ma C., Qiao Y., Yao H. (2016). Thermal Degradation of PVC: A Review. Waste Manag..

